# Acute Myopericarditis after the Second Dose of mRNA COVID-19 Vaccine Mimicking Acute Coronary Syndrome

**DOI:** 10.1155/2022/1600734

**Published:** 2022-08-09

**Authors:** Chris Pantsios, Zacharias-Alexandros Anyfantakis, Andrew Xanthopoulos, Ioannis Leventis, Spyridon Skoularigkis, Apostolos Dimos, Angeliki Bourazana, Michail Papamichalis, Grigorios Giamouzis, Filippos Triposkiadis, John Skoularigis

**Affiliations:** Department of Cardiology, General University Hospital of Larissa, Larissa, Greece

## Abstract

Myocarditis is a rare adverse event of vaccination. Recently, mRNA vaccines for COVID-19 have been reported to correlate with myocarditis, specifically in adolescents and young men. We report a rare case of a 50-year-old man who presented with symptoms of myocardial infarction 3 days after the second dose of vaccination for COVID-19. Cardiac magnetic resonance (CMR) imaging revealed acute myopericarditis. Clinicians should be aware of that rare side effect of mRNA vaccines for COVID-19 that can affect not only younger recipients but also middle-aged patients presenting with symptoms mimicking acute coronary syndrome.

## 1. Background

Myocarditis is an inflammatory cardiac disease induced by viruses, bacteria, protozoa, and fungi [1]. Severe acute respiratory syndrome coronavirus (SARS-CoV) and SARS-CoV-2 can trigger myocarditis either by direct cardiac injury or indirectly, due to host's immune response [2]. At the same time, myocarditis can be induced by a wide variety of toxic substances, drugs, and vaccines [3]. Postimmunization myocarditis is a known but rare adverse event following some vaccinations, particularly for smallpox [4] but also for influenza, tetanus, and hepatitis B [5]. Although there is a growing literature associating myocarditis with mRNA COVID-19 vaccines, reports are rather rare [6–8].

The clinical manifestations of myocarditis are highly variable and sometimes can mimic acute myocardial infarction, making diagnosis challenging [9].

We present a 50-year-old patient diagnosed with myopericarditis, the term for diagnosis of both myocardial and pericardial inflammation, 3 days after vaccination for COVID-19 with BNT 162b2 vaccine, presenting with symptoms of acute coronary syndrome.

## 2. Case

A 50-year-old man presented at the emergency department (ED) complaining for retrosternal, constrictive chest pain that awakened him up 2 hours before his arrival to our hospital. Three days before he received the second dose of vaccination for COVID-19 with BNT 162b2 vaccine and the same day, he reported low fever (37.5°C) and diarrhea.

The patient was tested negative at nasopharyngeal swab testing for SARS-CoV-2 PCR, as required prior to hospital admission. His chest pain resolved spontaneously within 6 h of his admission, lasting 8 hours in total.

Our patient did not report past medical history, neither risk factors for CAD, with the exception of a positive family history for premature CAD (his father suffered an acute myocardial infarction at the age of 45 years). He denied any symptom of infectious disease prior to the second dose of vaccination.

**Figure 1:**
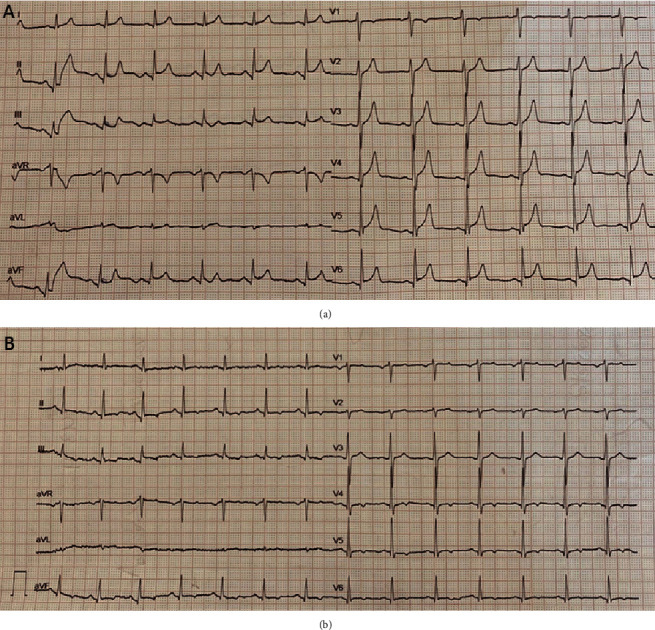
ECG at presentation (a) and at day 4 after admission (b). (a) ST elevation in inferior and lateral leads with concave up and PR segment depression can be noticed. (b) ST resolution and T wave inversion in inferior and lateral leads.

Clinical examination was normal. The patient was afebrile, with HR of 75 bpm and normal blood pressure (130/80 mmHg). Oxygen saturation was 99% on room air. Auscultation of the heart and lungs was normal. Electrocardiogram (ECG) in the ED showed ST segment elevation in inferior and lateral leads, pointing inferolateral wall involvement, with concave up and PR segment depression in the same leads (Figure 1(a)).

Troponin T was elevated at admission (0.088 *μ*g/L) and peaked at 0.71 *μ*g/L the next day (normal value < 0.017 *μ*g/L). Creatine kinase was also elevated (345 U/L, normal value < 198 U/L).

**Figure 2:**
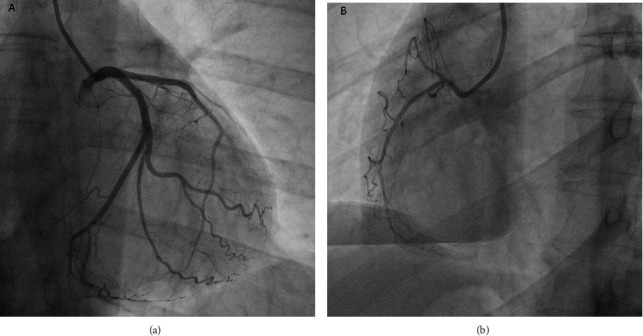
Coronary angiogram ((a) left coronary artery and (b) right coronary artery) demonstrating normal coronary arteries.

Clinical presentation, electrocardiographic changes, and elevated cardiac markers led the patient to the catheterization lab. Thirty minutes after the patient's arrival at the emergency department, an emergent coronary angiography was performed to rule out acute coronary syndrome revealing normal epicardial arteries (Figure 2). As myocarditis emerged as the most likely diagnosis, antithrombotic and antiplatelet therapy was discontinued and the patient remained in the coronary care unit (CCU) for monitoring. During his first day of hospitalization, he reported two episodes of mild chest pain, of 30 min duration each, with no ECG changes and spontaneous resolution.

**Figure 3:**
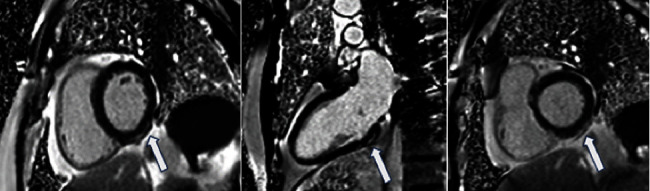
Postcontrast images of cardiac magnetic resonance (6 days after the patient's admission) in short and long views showing subepicardial enhancement in the basal and midinferior and inferolateral wall of the left ventricle (white arrows).

The echocardiogram was normal with neither regional wall motion abnormalities nor pericardial effusion. CMR was performed 6 days after the admission (SIEMENS AVANTO 1.5 T). It demonstrated normal left ventricular size and ejection fraction (60%), postgadolinium diffuse subepicardial enhancement in the inferior and inferolateral LV wall, and increase in T1 (1159 s) and T2 (57ms) time in comparison with the rest myocardium, confirming the diagnosis of myocarditis (revised Lake Louise criteria) [10]. Thickening and enhancement of the adjacent pericardium were also shown (Figure 3).

Due to mild in-hospital symptomatology and absence of heart failure, the patient did not receive specific treatment for myocarditis or pericarditis. During his six-day hospitalization, he remained well appearing, hemodynamically stable, and in sinus rhythm. Viral studies for related to myocarditis viruses were performed and did not reveal IgM antibodies nor fourfold increase in IgG antibodies. The workup concerned Epstein-Barr virus, cytomegalovirus, adenovirus, enterovirus (coxsackie A, coxsackie B1), hepatitis B and C viruses, and human herpes 1 and 2 viruses.

Cardiac troponin T and creatine kinase progressively decreased, and ECG showed ST segment resolution with T wave inversion (Figure 1(b)). The patient was discharged home with the recommendation to avoid intense physical activity for three to six months.

**Figure 4:**
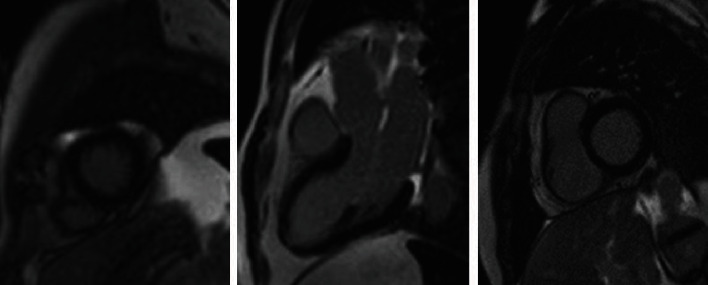
Postcontrast images of cardiac magnetic resonance (at 6 months) in short and long views showing minimal subepicardial enhancement in the basal inferior and inferolateral wall of the left ventricle.

Six months after his admission, CMR was repeated and revealed normal left ventricular size and function and mild postgadolinium subepicardial enhancement in the inferior and inferolateral LV wall, compatible with healed myocarditis (Figure 4). T1 time was decreased to 1036 ms (from 1159 ms) showing inflammation remission. The patient remains well appearing and asymptomatic.

A written informed consent was provided by the patient for the publication.

## 3. Discussion

Our patient meets the criteria for confirmed acute myocarditis (clinical symptoms and pathologic troponin levels and CMR findings consistent with myocarditis and no other identifiable cause of the symptoms and findings) [11, 12]. He also meets criteria for acute pericarditis (acute chest pain and compatible ECG changes). This case is rare as until 11^th^ of June 2021, only 50 cases of people over 50 years have been reported to the US Vaccine Adverse Events Reporting System (VAERS) after over 34 million doses of mRNA COVID-19 vaccines administered [11].

One potential cause of COVID-19 vaccine myocarditis may be the pathologic immune response to vaccine's mRNA. Certain predisposed individuals detect mRNA molecules of vaccine as antigens and activate immunologic pathways and proinflammatory cascades that may play a role in myocarditis [13]. Another potential mechanism may be the molecular mimicry between the spike protein of SARS-CoV-2 and self-antigens. Antibodies against SARS-CoV-2 spike glycoproteins have been shown to cross-react with structurally similar human peptide proteins, including *α*-myosin [14].

The incidence of myocarditis after vaccination is very low. The estimated incidence of at least one dose of the BNT162b2 mRNA vaccine was reported to be 2.13 cases per 100,000 vaccinated persons in the first 42 days after receipt [15]. The CDC COVID-19 Vaccine Task Force has reported until 11^th^ of June 2021 a rate of myopericarditis ranging from 18.1/1.000.000 doses in adolescents of 12-17 y. to 1.7/1.000.000 doses in people older than 50 years [11].

On the contrary, nearly 2.3% of highly fit athletes with mild COVID-19 infection have evidence of myocarditis on CMR [16] and recently, in a second study, 0.6% of professional athletes have demonstrated detectable inflammatory heart disease on CMR [17]. While the actual prevalence of myocarditis in COVID-19 patients remains unknown, it is estimated that cardiac injury after COVID-19 infection is as high as 19-28% and may reach 60% in seriously ill patients [18]. It is obvious that there is a significantly higher risk of cardiac involvement from COVID-19 infection compared to COVID-19 vaccination.

Given that the majority of cases have a mild clinical course, COVID-19 vaccination should remain the cornerstone for population immunity. It is important to note that a causal relation between the mRNA-COVID-19 vaccine and myocardial inflammation has not been proven yet.

The retrosternal, constrictive chest pain which made our patient seek medical care subsided a few minutes after his arrival to the ED. His symptoms thereafter—during his hospitalization—were mild. Patients with myocarditis mimicking AMI have a favorable long-term prognosis [19]. It is noteworthy that after six months, our patient remains asymptomatic.

Recognition of vaccine-associated myocarditis is important, as diagnosis impacts management, recommendations for exercise, and surveillance. Whether vaccination schedule needs to be modified for patients who have a history of myocarditis, how should these cases be managed, and what type of cardiac follow-up would be appropriate remain to be answered.

Clinicians should be aware that myopericarditis after COVID-19 vaccination may not exclusively affect younger individuals but it should be also considered in middle-aged patients presenting with acute coronary syndrome symptoms.

## 4. Conclusion

Myocarditis is a rare adverse event of mRNA vaccines for COVID-19. Clinicians should be aware that myopericarditis can affect not only younger vaccine recipients but also middle-aged patients presenting with symptoms mimicking acute coronary syndrome. Given that there is a significantly higher risk of cardiac involvement from COVID-19 infection compared to mRNA vaccines, vaccination should remain the cornerstone for population immunity.
